# Increasing Antiretroviral Adherence for HIV-Positive African Americans (Project Rise): A Treatment Education Intervention Protocol

**DOI:** 10.2196/resprot.5245

**Published:** 2016-03-29

**Authors:** Glenn J Wagner, Laura M Bogart, Matt G Mutchler, Bryce McDavitt, Kieta D Mutepfa, Brian Risley

**Affiliations:** ^1^ RAND Corporation Health Unit Santa Monica, CA United States; ^2^ Department of Sociology California State University Dominguez Hills CA United States; ^3^ AIDS Project Los Angeles CA United States; ^4^ Center for AIDS Research and Education David Geffen School of Medicine University of California Los Angeles CA United States; ^5^ Men's Health Foundation West Hollywood, CA United States

**Keywords:** antiretroviral treatment, adherence, patient compliance, HIV, African Americans, treatment education, intervention

## Abstract

**Background:**

HIV-positive African Americans have been shown to have lower adherence to antiretroviral therapy (ART) than those of other races/ethnicities, yet adherence interventions have rarely been tailored to the needs of this population.

**Objective:**

We developed and will evaluate a treatment education adherence intervention (called Rise) that was culturally adapted to address the needs of African Americans living with HIV.

**Methods:**

This randomized controlled trial will examine the effects of the Rise intervention on ART adherence and HIV viral load. African Americans on ART who report adherence problems will be recruited from the community and randomly assigned to receive the intervention or usual care for 6 months. The intervention consists of 6-10 individual counseling sessions, with more sessions provided to those who demonstrate lower adherence. Primary outcomes include adherence as monitored continuously with Medication Event Monitoring Systems (MEMS) caps, and viral load data received from the participant’s medical provider. Survey assessments will be administered at baseline and month 6.

**Results:**

The trial is ongoing.

**Conclusions:**

If effective, the Rise intervention will provide community-based organizations with an intervention tailored to address the needs of African Americans for promoting optimal ART adherence and HIV clinical outcomes.

**Trial Registration:**

Clinicaltrials.gov NCT01350544; https://clinicaltrials.gov/ct2/show/NCT01350544 (Archived by WebCite at http://www.webcitation.org/6fjqqnmn0).

## Introduction

Compared to Whites living with HIV, African Americans with HIV have lower engagement and retention in care [1-3], are less likely to receive antiretroviral treatment (ART) [4,5], and are less likely to adhere to treatment long enough for it to be effective [4-9]—all of which may contribute to disparities in survival [10-13].

Research has identified culturally specific determinants of ART adherence among African Americans, including stigma, medical mistrust, and HIV-related misconceptions (eg, that ART is poison) [7,8,14,15]. Research also suggests that social conditions such as poverty, health care factors (eg, provider behaviors contributing to mistrust), and psychosocial issues such as mental health need to be addressed in interventions focused on improving HIV-positive African Americans’ health behaviors and ultimately their health outcomes [16-19]. However, interventions to improve adherence have rarely been culturally tailored to the needs of African Americans, which may partially explain the lack of robust effects observed in reviews of ART adherence intervention trials that contain large numbers of African American participants [20-22].

Treatment education programs have been used by AIDS service organizations (ASOs) across the United States to facilitate adherence to HIV care through client-centered one-on-one counseling. Treatment education counselors possess specialized HIV treatment knowledge, target structural issues in health care and social conditions in clients’ lives, counsel clients to overcome adherence barriers, recommend changes in treatment and/or providers (if needed), and refer clients to mental health, substance abuse, and social services (eg, for housing). Treatment education is particularly appropriate for patients who may be mistrustful of providers, including African Americans, because it can be conducted outside of the medical system, and teaches patients self-advocacy in health care.

To our knowledge, the efficacy of treatment education has not been evaluated in a randomized controlled trial (RCT). In a prior process evaluation of the longstanding treatment education program at AIDS Project Los Angeles (APLA), clients in treatment education showed higher adherence and engagement in care, greater perceived ART efficacy, and fewer unmet social service needs, compared to clients not receiving treatment education [23]. Furthermore, treatment education was viewed by medical providers, treatment education counselors, and patients as increasing understanding about treatment, support adherence, and improving patient-provider relationships [24].

**Figure 1:**
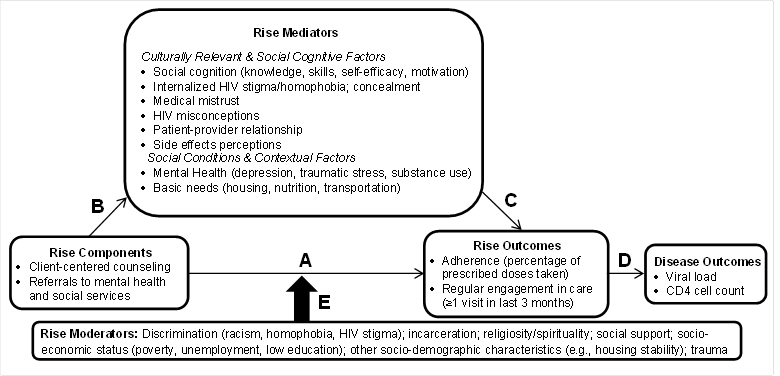
Conceptual model of Rise, a culturally tailored treatment education program.

We developed the structured, culturally tailored treatment education intervention *Rise*, named by the community-academic team after the Maya Angelou poem [25] “Still I Rise,” which emphasizes resilience in Black communities. We are evaluating Rise’s effects on adherence among African Americans in an RCT, the protocol for which is described in this paper. Rise is built around the core components of the treatment education program being implemented by APLA, which include a needs assessment, individual counseling, and referrals as required. These components are proposed to synergistically influence sociocultural factors that predict adherence (see Figure 1). Client-centered counseling builds treatment knowledge and adherence skills, self-efficacy, and motivation (key adherence predictors) [26-28]. Given robust relationships of homelessness, depression, traumatic stress, and substance use with adherence [29-30], referrals to mental health/social services can improve adherence by decreasing unmet needs. Rise also addresses culturally relevant coping strategies (eg, medical mistrust) that arise in response to factors such as discrimination and stigma, which can undermine health care access and use in Black communities [31,32].

This paper describes in detail the methodological protocol, intervention content, and structure of Rise. If effective in improving ART adherence and virologic suppression, Rise will provide the field with one of the first ART adherence interventions tailored specifically to address the needs of African Americans.

## Methods

### Study Design

The study is an RCT to compare Rise, a culturally tailored and structured adherence counseling intervention, with usual care (received from participants’ primary care providers, with no added intervention), among African Americans living with HIV. To determine effects on ART adherence, participants complete audio computer-assisted self-interviews (ACASI) at baseline and 3- and 6-months postbaseline to assess self-reported adherence, and interviewers check in with participants at 1-, 2-, 4-, and 5-months postbaseline to download electronically monitored adherence data, collected via the Medication Events Monitoring System (MEMS) described below, and to update contact information. Participants are paid $30 at baseline and 3- and 6-month follow-ups, and $10 for each check-in (1, 2, 4, & 5 months postbaseline). If all assessments are completed, participants receive a $20 bonus. Participants also receive a second $20 bonus for updating primary contact information during the study (eg, address, phone, email). Participants receive a snack and $10 per intervention session for transportation costs. The Human Subjects Protection Committee of the RAND Corporation and the Institutional Review Board of California State University, Dominguez Hills approved the study protocol, and a Certificate of Confidentiality was obtained from the National Institutes of Health. The trial is registered with the National Institutes of Health sponsored clinical trials registry and assigned the identifier NCT01350544 (date released: 5/26/2011).

### Study Setting

The study is being conducted at AIDS Project Los Angeles (APLA), the largest ASO in Los Angeles County. APLA provides direct, bilingual services (eg, treatment education, case management, substance use and mental health treatment, housing and food assistance) to ~10,000 men, women, and children with HIV in Los Angeles county annually. The majority of APLA clients are ethnic or racial minorities, with about one-quarter being African American, and nearly 90% male, which is consistent with the racial/ethnic and gender distribution of people living with HIV in Los Angeles.

The study team represents a partnership among researchers, APLA program staff, and HIV community stakeholders including a community advisory board (CAB) composed of clients living with HIV (most of whom are African American), APLA staff, and HIV service agency and staff. This has played a pivotal role in the study design and suggested directions for the intervention. Throughout the ongoing RCT, CAB members have been integral in reviewing project methodology and materials, evaluating study progress, and monitoring program implementation; when the results are available, they will also help with interpreting study data. Eliciting the feedback of the CAB members is facilitated by in-person CAB meetings that are convened at key junctures of the study, about two to three times during the course of each year of the study. CAB members are given a small honorarium for their time ($50 per meeting).

###  Participants

Eligibility criteria include the following: (1) age ≥18 years; (2) self-identification as African American or Black (if mixed race, primarily identify as African American); (3) on ART; (4) reported any adherence problems (ie, missed ≥1 ART dose in the past month, less than 100% adherence in the past month, sometimes stopped ART if they felt worse, and/or any missed doses during the past weekend); (5) not currently participating in another treatment adherence intervention; and (6) willing to use an electronic monitoring cap to monitor adherence to one of their antiretrovirals. Individuals who have received treatment education services (from APLA or elsewhere) in the last 6 months are excluded. Participants are recruited through APLA and other community-based organizations (eg, SPECTRUM, OASIS) via public presentations of the study and flyers, referrals from providers, and radio and print advertisements.

### Treatment Advocacy Intervention (Rise)

The 24-week intervention consists of 4 individual, weekly, 60-minute adherence counseling sessions and 1 group HIV education session during the first month, which comprises the core adherence training phase. This is followed by a maintenance phase, in which participants receive booster sessions at weeks 12 and 20, plus up to 4 additional sessions depending on adherence performance (ie, sessions added if <90% of prescribed doses taken in past month). In total, participants receive between 6 and 10 individual sessions. Table 1 describes the intervention components, goals, and activities of each session, and the mediators of intervention effects that are addressed by these activities. Table 2 provides an outline of the exercise content within each session.

**Table 1:** Rise session components, goals, activities, and proposed mediators addressed.

Rise Component/Goal	Individual Session	Counselor Activity	Mediator Addressed
*Client-centered Counseling*: Improve motivation for adherence and care engagement through use of MI^a^ style and provision of strategies to reduce barriers	1	After introducing the program, assess client’s knowledge and provide education on HIV, ART^b^, adherence, and health disparities; discuss culturally relevant stressors (eg, discrimination) that can affect health and health behaviors	Improved adherence cognitions (knowledge, skills, self-efficacy, motivation); lower HIV misconceptions & medical mistrust
	All	Review electronic adherence data, offer encouragement and reinforce good adherence; in all sessions after Session 1, assess changes in engagement in care and adherence	Improved adherence cognitions
	All	Discuss attitudes and beliefs, including culturally relevant health beliefs; encourage positive attitudes toward treatment	Lower HIV misconceptions & mistrust
	All	Assess support availability, and level of stigma and awareness of HIV status in the network; problem solve ways to increase support	Greater HIV disclosure
	2-10	Use steps for problem solving to identify barriers to engagement in care and adherence, and plan solutions	Improved adherence cognitions
	2-10	Discuss integration of medication and appointments into daily routine	Improved adherence cognitions
	2-10	Discuss strategies to reduce/manage medication side effects	Reduction in perceived side effects
	All	Develop (in Session 1) and review (in other sessions) Individual Service Plan (ISP) of short- and long-term goals; proposed timeline and outcomes; and client and medical provider tasks	Improved adherence cognitions
*Mental Health/Social Services Referrals*: Link to services to improve mental health/reduce unmet needs, to support adherence and care engagement	All	Conduct needs assessment and address problem areas, including social and structural issues (eg, substance abuse/mental health, housing/nutrition) that influence adherence; provide needed referrals	Fewer unmet basic subsistence needs; fewer/less severe mental health symptoms (depression, traumatic stress); lower substance use
*Facilitated Group Education*: Provide basic education; use group dynamic to increase treatment motivation and in turn, adherence and care engagement	Between 1 & 4	Provide basic information on prevention, transmission, progression, treatment, and adherence; encourage group discussion and address any HIV-related misconceptions	Improved adherence cognitions, lower HIV misconceptions and medical mistrust
	Between 1 & 4	Present information on HIV and adherence disparities, and racism, homophobia, and HIV stigma as contributors to disparities; encourage sharing of health care experiences	Lower HIV misconceptions, medical mistrust, and internalized stigma
	Between 1 & 4	Promote collective responsibility to motivate adherence (eg, stay healthy to keep Black communities strong)	Improved adherence cognitions

^a^ motivational interviewing

^b^ antiretroviral therapy

**Table 2:** Outline of intervention session content.

Session 1 (Week 00)	1. Check in with the client to begin to build rapport. 2. Introduce the intervention and its goals. 3. Review client’s history of HIV diagnosis and ART, and relationship with provider. 4. Provide education about goals of ART and importance of adherence. 5. Assess client’s attitudes towards treatment and adherence. 6. Conduct needs assessment. 7. Develop client’s Individual Service Plan (ISP).
Session 2 (Week 2)	1. Check in with client to build rapport. 2. Review progress with ISP since last session and follow up on referrals made. 3. Review adherence using the MEMS print out. 4. Identify barriers to adherence. 5. Introduce problem solving steps. 6. Apply problem solving steps to one barrier. 7. Tailor regimen and adherence to daily routine cues. 8. End with a statement of affirmation.
Session 3 (Week 4)	1. Check in with client to build rapport. 2. Review progress with ISP since last session and follow up on referrals made. 3. Review adherence since last session using the MEMS print out. 4. Identify barriers to adherence. 5. Apply problem solving steps to one barrier. 6. Enhance social support for adherence. 7. Revisit attitudes towards treatment and adherence. 8. Discuss plans to interact with client’s HIV provider. 9. End with a statement of affirmation.
Maintenance Module A/B (Week 12/20)	1. Check in with client to build rapport. 2. Review progress with ISP since last session and follow up on referrals made. 3. Review adherence over past month using the MEMS print out. 4. Identify barriers to adherence. 5. Apply problem solving steps to one barrier. 6. Enhance social support for adherence. 7. Revisit attitudes toward treatment and adherence. 8. Review status of doctor-patient relationship. 9. End with a statement of affirmation.
Last Session Add-Ons	1. Instill confidence and self-efficacy for long-term adherence. 2. Set goals related to adherence over the next few months.

#### Core Adherence Training Phase

##### Session 1 (Week 1)

In this first session, the counselor provides information about the importance of adherence and consequences of missed doses, provides education about viral load and drug resistance, and explains the connection between dosing schedules and viral suppression. A motivational interviewing (MI) style [33] is used to help clients develop or strengthen positive attitudes toward treatment and adherence. We do not attempt to adhere strictly to the use of MI, but the counselor is trained to ask open questions, to use reflective listening, and to motivate change by highlighting discrepancies between behavior or thoughts and stated health goals, as well as respecting client autonomy. Following a needs assessment, the counselor provides referrals for any unmet basic needs (housing, food, transportation) and mental health issues (depression, traumatic stress, substance use).

To address culturally relevant factors, the counselor acknowledges health care discrimination and health disparities, and shares reasons for medical mistrust and HIV misconceptions in Black communities. The counselor encourages the client to share any experiences and perceptions they may have in this regard. To enhance instrumental (eg, finding transportation to clinic) and emotional (eg, encouraging/reinforcing adherence) support, the counselor assesses the availability of social supports to the client (eg, whether individuals in clients’ social networks are aware of their HIV status). The counselor explores with the client whether HIV disclosure is an option for obtaining social support, but in the context of reviewing both possible benefits as well as risks to disclosure. If the client reports HIV-related misconceptions, the counselor supplies accurate information to dispel the misconceptions.

To diminish mistrust, the counselor works to establish a collaborative relationship in which the client can experience the counselor as an equal, identify commonalities, and be regarded as a whole person [34]. This may involve using nontechnical or vernacular language when appropriate, and allowing time for clients to discuss issues not directly related to the intervention aims (to demonstrate investment in the client as a person). The counselor maintains a nonjudgmental attitude, to help clients feel safe revealing gaps in their HIV treatment knowledge and reasons for missed doses. At times, the counselor may also reflect on his or her own experiences. For example, when addressing conspiracy theories about HIV, the counselor may share if he or she originally held similar beliefs, and how these views changed through learning more about the relevant research. Sharing these more personal experiences helps the client view the counselor as a “genuine” person who is speaking based on his or her authentic values and feelings—rather than merely performing a professional role. This is also intended to reduce mistrust by modeling critical thinking and receptivity to scientifically based information. 

Session 1 closes with the development (in partnership with the client) of an Individual Service Plan (ISP) of short- and long-term goals (eg, following up on referrals, engaging a friend to seek support for adherence), which is revisited and updated in each successive session.

##### Sessions 2-4 (Weeks 2-4)

These sessions are similar in content to Session 1, but with greater targeting of barriers to medication adherence and retention in care. Together with the client, the counselor reviews adherence (using the printout from the MEMS software, which provides adherence summary statistics and graphic depictions of patterns of doses taken and missed) and attendance at scheduled doctor’s visits since the last session. The client identifies barriers that may contribute to missed appointments and nonadherence, such as lack of accurate treatment information (or belief in misconceptions), low motivation, presence of or concern about treatment side effects, mental health problems or substance use, internalized stigma, and medical mistrust and/or negative experiences with health care (eg, discrimination). Stages of problem solving are reviewed: defining the problem, deciding on a goal, generating possible solutions, selecting a potential solution, planning the solution’s implementation, and evaluating the solution’s effectiveness (at the subsequent session). 

The counselor identifies contextual cues that influence pill-taking (ie, what occurred immediately before and after a missed dose/appointment), to derive strategies for managing and controlling cues. Clients describe daily routines and together with the counselor determine optimal ways to integrate medication into these routines. Doses are connected with routine daily activities that can serve as reminder triggers for taking medications. Strategies for coping with and reducing side effects are discussed. The counselor assesses whether clients followed up on referrals, offers new referrals as needed, and evaluates progress toward ISP goals. Within these early sessions, the counselor assesses the client’s relationship with their HIV care provider in terms of satisfaction with care received and level of support from and trust in their provider. With consent from the client, the counselor contacts the provider via phone or email to inform them that the client is participating in the program and working with the counselor over the next 6 months to support the client’s adherence and care retention.

##### Group HIV Session

Before Session 4, clients are offered an hour-long HIV education group, facilitated by the counselor, along with other study participants who are receiving the intervention. The counselor provides basic HIV and ART information from APLA’s standard educational forum, facilitates discussions of how stigma, discrimination, and medical mistrust contribute to health disparities in Black communities, and encourages participants to share experiences with and learn from each other.

#### Maintenance Adherence Training Phase

##### Booster Sessions 5 & 6 (Weeks 12, 20)

The goal of the maintenance phase is to help clients sustain optimal adherence. Clients who achieve ≥90% adherence (as measured by the MEMS cap) during the 2 weeks preceding Weeks 12 and 20 receive a session only at those weeks; others receive up to two biweekly added sessions at each time point. For example, if adherence from Weeks 10-12 is ≥90%, the client receives a session at Week 12, and then returns again at Week 20 for the next booster session. If the client’s adherence from Weeks 10-12 is <90%, the client receives maintenance sessions at Weeks 12 and 14, and if adherence from Weeks 12-14 is <90%, then s/he receives another session at Week 16. All clients return at Week 20, but only clients with adherence <90% during Weeks 18-20 return at Week 22, and those with adherence <90% during Weeks 20-22 have another session at Week 24. We used 90% adherence as the cutoff for determining good adherence based on research suggesting that this level of adherence was associated with optimal treatment response and virologic suppression [35). This dose regulation (of 0-4 extra sessions) is intended to promote efficient use of limited community resources by varying intensity depending on client need. Maintenance sessions are similar to the initial core sessions, with continued emphasis on identification and resolution of barriers, use of motivational enhancement techniques to improve attitudes and motivation related to adherence, side effect management, and monitoring of adherence-related social support and self-efficacy, and medical mistrust.

#### Intervention Counselor Training, Fidelity Monitoring, and Supervision

Counselor training and ongoing supervision is used to ensure consistency in intervention implementation and monitor fidelity to the intervention protocol. The counselor training includes instruction on basic HIV disease information, the importance of protecting confidentiality and complying with Health Insurance Portability and Accountability Act (HIPAA) regulations, crisis intervention, referral resources, cultural and social issues that can influence treatment adherence among African Americans, and mental health and substance abuse assessment. The counselor is trained to establish rapport and effective working relationships with clients and medical providers, and locate community resources, referrals, and linkages. The counselor is provided with and encouraged to further develop a comprehensive referral list to HIV-related and auxiliary services (eg, housing, mental health). All counseling sessions are audio-recorded, with consent from the client. The supervisor listens to all recorded sessions of the first 2 clients receiving the intervention, and then all recorded sessions of every 5^th^ participant. This serves as the basis for provision of feedback to the counselor during biweekly supervision. Supervision also provides a time for the counselor to discuss challenging cases and review treatment plans for newly enrolled participants.

### Measures

Below are descriptions of the primary outcomes to be assessed (ART adherence, HIV care retention, and HIV clinical outcomes). The survey assessment includes measures of potential mediators of intervention effects (eg, attitudes and beliefs related to HIV, ART and adherence, stigma, discrimination, mental health and substance use), and potential moderators (eg, socio-demographic characteristics); however, these are not described in detail here.

#### ART Adherence

Adherence to one antiretroviral (with the most complex regimen) is measured electronically and continuously throughout the study using the MEMS. Clients are instructed to remove one dose at the time that they plan to ingest the dose, and to refill the bottle after the last pill is removed. Participants do not always follow these instructions (eg, removing multiple doses at once or “pocketing”); thus, we assess their actual use of the cap via self-report in the past 2 weeks at each primary assessment, and then adjust the number of prescribed does taken during this period using this information. This adjustment method has been validated [35] and shown to strengthen the relationship between adherence and viral load. MEMS software calculates several adherence parameters including the percentage of scheduled doses taken, and yields printouts of adherence data and charts that depict adherence patterns (which are used to facilitate the adherence counseling sessions). We also ask participants to self-report the number of doses missed in the last week and percentage of prescribed medications taken in the last month.

#### HIV Care Retention

Clients are asked to report the number of appointments scheduled, attended, and missed with their HIV care provider in the past 6 months.

#### HIV Clinical Outcomes

Clients are asked to provide HIPAA-compliant informed consent for access to medical records data, which we are using to request the last 2 HIV viral load and CD4 count assay results from providers.

### Statistical Analysis

To examine the effects of Rise on the primary outcomes, we will use ordinary least squares regression for continuous outcomes (mean adherence; change in viral load and CD4) and logistic regression for dichotomous outcomes (adherence >90%; undetectable viral load). Group assignment (intervention or control) will be entered in the model as a dummy variable, together with the baseline value of the outcome and covariates. For comparability across outcomes, we will choose a single set of covariates across models, from variables hypothesized based on theory and prior research to predict outcomes, or for which we find support for hypothesized relationships in bivariate baseline analyses. We will drop predictors for which there are large correlations to avoid collinearity. We will use an intent-to-treat approach as the primary analysis, grouping participants by the condition to which they were randomized, regardless of level of participation or study completion; a secondary analysis will be performed with study completers only.

## Results

The project was funded in 2012 and enrollment was completed in 2015. Data analysis is currently underway and the first results are expected to be submitted for publication in 2016.

## Discussion

This study will be one of the first to evaluate an HIV treatment adherence intervention for African Americans that integrates culturally relevant factors with basic components of treatment education, an established program commonly found in AIDS service organizations. Although many HIV adherence intervention trials have included a substantial number of African American participants, few were developed specifically for African Americans and take into account their unique cultural context. Interventions to improve African Americans’ adherence have rarely considered cultural and social determinants of health behavior, including medical mistrust, HIV misconceptions, and experiences with discrimination [36].

Although not specific to adherence, meta-analyses indicate that most HIV-prevention interventions for women are less consistently effective for African Americans [37], and interventions addressing culture tend to have larger effects [38]. Further, most adherence interventions are conducted in HIV primary care clinics [39]. For greater success, adherence programs need to take into account African Americans’ mistrust of “outsiders” (which limits acceptance of HIV care), openly acknowledge factors such as racism that undermine health care in Black communities [40,41], and address mistrust and stigma as coping strategies that arise in response to racism and oppression. Moreover, given African Americans’ high levels of medical mistrust, community-based programs such as Rise may have greater reach and effectiveness than those in clinics.

In sum, Rise integrates current practice with science by examining whether an untested yet sustained community program is effective. Our program also challenges a growing policy movement in HIV clinical care that seeks to engage patients for social services via medical providers (within the confines of the medical system) rather than community settings. Rise’s community-based paradigm provides an alternative source of treatment support, which may be critical for African Americans who exhibit high levels of mistrust and low adherence, and for whom no culturally relevant rigorously tested adherence intervention exists. In this way, Rise is a holistic program that includes client-centered treatment education to intrinsically motivate treatment behavior changes, and referrals to auxiliary services targeting unmet social service needs to ultimately improve health outcomes.

## Appendix

Multimedia Appendix 1 Summary statement from NIH review.

## Abbreviations

ACASI: audio computer-assisted self-interviews
AIDS: acquired immune deficiency syndrome
APLA: AIDS Project Los Angeles
ART: antiretroviral therapy
ASOs: AIDS service organizations
CAB: community advisory board
HIPAA: Health Insurance Portability and Accountability Act
HIV: human immunodeficiency virus
ISP: Individual Service Plan
MEMS: Medication Event Monitoring Systems
MI: motivational interviewing
RCT: randomized controlled trial
