# Supplementary material for: Increasing Antiretroviral Adherence for HIV-Positive African Americans (Project Rise): A Treatment Education Intervention Protocol
Source: JMIR Res Protoc. 2016 Mar 29;5(1):e45. doi: 10.2196/resprot.5245 (PMC4829729; doi:10.2196/resprot.5245)
Supplement: Multimedia Appendix 1 [file resprot_v5i1e45_app1.PDF]

SUMMARY STATEMENT
(Privileged Communication)

PROGRAM CONTACT:
Jennifer Alvidrez
301-594-9567
alvidrezjl@mail.nih.gov

Release Date: 02/01/2011

Application Number: 1 R01 MD006058-01A1

Principal Investigator

BOGART, LAURA M PHD

Applicant Organization: CHILDREN'S HOSPITAL BOSTON

Review Group: ZMD1 MLS (01)
National Center on Minority Health and Health Disparities Special Emphasis Panel R01

Meeting Date: 12/15/2010
Council: JAN 2011
Requested Start: 03/01/2011
RFA/PA: MD11-001
PCC: R01
Dual IC(s): NR

Project Title: Treatment Advocacy Intervention for HIV-Postive African Americans

SRG Action: Impact/Priority Score: 17
Human Subjects: 30-Human subjects involved - Certified, no SRG concerns
Animal Subjects: 10-No live vertebrate animals involved for competing appl.
Gender: 1A-Both genders, scientifically acceptable
Minority: 2A-Only minorities, scientifically acceptable
Children: 1A-Both Children and Adults, scientifically acceptable
Clinical Research - not NIH-defined Phase III Trial

Project Year	Direct Costs Requested	Estimated Total Cost
1	250,000	401,651
2	250,000	401,651
3	250,000	401,651
4	250,000	401,651
5	250,000	401,651
TOTAL	1,250,000	2,008,256

ADMINISTRATIVE BUDGET NOTE: The budget shown is the requested budget and has not been adjusted to reflect any recommendations made by reviewers. If an award is planned, the costs will be calculated by Institute grants management staff based on the recommendations outlined below in the COMMITTEE BUDGET RECOMMENDATIONS section.

1R01MD006058-01A1 BOGART, LAURA

(**NOTE:** The critiques below were prepared by the reviewers assigned to this application. These commentaries do not necessarily reflect the position of the authors at the close of the group discussion, nor the final majority opinion of the group, although reviewers are asked to amend their critiques if their position changed during the discussion. The resume and summary of discussion, together with the sections at the end of the summary statement, which summarize the committee's final opinion on the use of human subjects, the inclusion of women, minorities, and children, the use of vertebrate animals, and budget are the authoritative representation of the final outcome of group discussion. If there is any discrepancy between the peer reviewers' commentaries and the numerical score on the face page of this summary statement, the numerical score should be considered the most accurate representation of the final outcome of the group discussion.)

HUMAN SUBJECT CODE = 30

OVERALL EVALUATION: (Priority Score: 17)

RESUME AND SUMMARY OF DISCUSSION: This is an exceptional application by Dr. Laura Bogart from Children's Hospital Boston, in response to RFA-MD-11-001 NIMHD Health Disparities Research (R01). The PI proposes to evaluate an already created culturally relevant Treatment Advocacy (TA) intervention and its impact on HIV treatment in African Americans, using a randomized controlled trial. The applicant proposes to assess the effect of the intervention and examine the mediators, moderators and determinants explaining adherence or non-adherence to treatment. The strengths of the proposal are that it is an extremely significant project and culturally relevant for the targeted population. The application is found to be responsive to the comments from the previous application. The investigators have described a perfectly tailored intervention and have great experience working with the target population. The social and cultural issues to be studied are innovative and well tied to the intervention. The recruitment from a community base affords the possibility of recruiting potential participants who may mistrust the health system. The environment is appropriate and strong letters of support from the communities accompanied the application. There are some concerns that detract from the level of enthusiasm. For example, the assessment is outdated for research desires and will need further detail. Overall, the level of enthusiasm among the panel members is very high and the application is rated at an Exceptional level judged against the balance of strengths and weaknesses.

DESCRIPTION (provided by applicant): Compared to other races/ethnicities, African Americans with HIV have lower levels of engagement in care, are less likely to be on antiretroviral treatment (ART), and are more likely to delay care and ART initiation; those on ART are less likely to be adherent at high enough levels for the treatment to be effective. We propose to test an innovative, culturally relevant treatment advocacy (TA) intervention for African Americans with HIV that targets social and cultural issues contributing to poor HIV treatment behaviors. TA, which has been sustained in many community organizations throughout the HIV epidemic, has never been systematically evaluated. TA facilitates patient navigation through the medical system and provides tailored HIV treatment education and client-centered counseling to improve adherence and engagement in care. TA targets social and contextual issues in healthcare and patients' lives by advocating to providers to improve patient-provider relationships, recommending changes in treatment and/or providers (if needed), and referring patients to mental health and social services. TA is particularly appropriate for African Americans with HIV, who may be mistrustful of providers: it can be conducted outside of the medical system in a safe, neutral community setting by individuals not associated with patients' healthcare. We developed a culturally relevant TA program that additionally discusses factors such as racism that undermine healthcare in Black communities, by acknowledging and directly addressing patients' medical mistrust and stigma as coping strategies that arise in response to oppression. The specific aims are to (1) conduct a randomized controlled trial to examine the effects of a culturally relevant TA program on adherence among African Americans with HIV; (2) identify culturally relevant mediators that explain the effects of treatment advocacy on antiretroviral treatment adherence among African Americans with HIV

(e.g., improved behavioral adherence skills, coping with stress/discrimination, mental health, and patient satisfaction; lower levels of HIV misconceptions, internalized HIV stigma/homophobia, medical mistrust, and substance use); and (3) explore culturally relevant moderators of the effects of treatment advocacy on antiretroviral treatment adherence among African Americans with HIV (e.g., discrimination, incarceration, poverty, social support, spirituality, trauma). A sample of 200 African Americans with HIV will be randomly assigned to a TA intervention or wait-list control group. Participants will complete surveys at screening, and at 3- and 6-months post-baseline, to assess pre-, intra-, and post- intervention effects on adherence.

PUBLIC HEALTH RELEVANCE: African Americans with HIV have lower levels of engagement in care and treatment adherence than do Whites with HIV, and the predictors of these behaviors differ by race/ethnicity; however, few culturally relevant interventions have been tested. We propose to conduct a randomized controlled trial (RCT) of an innovative, culturally relevant treatment advocacy (TA) intervention for African Americans with HIV that targets cultural and social issues contributing to health disparities. TA, which has been sustained in communities throughout the HIV epidemic but never been rigorously tested, facilitates medical system navigation and adherence through client-centered counseling and education; advocacy to providers; and referrals for social services.

CRITIQUE 1:

Significance	1
Investigator(s)	1
Innovation	1
Approach	2
Environment	1

OVERALL IMPACT:

Strengths

- Project will study an intervention for an important problem of HIV treatment adherence in a vulnerable population.
- PI and team are well qualified to conduct the project.
- The PI is committed to the reduction of health disparities.
- Strong institutional/agency support.
- The use of a culturally relevant framework where the intervention includes the use of MI, placement of the TA in a community setting, and counselors from the community is innovative.

Weaknesses

- CBPR principles to guide the proposed research process not identified.

SIGNIFICANCE:

Strengths

- This proposal includes a five year randomized clinical trial that will address a large racial and ethnic disparity issue relating to African Americans living with HIV lower levels of antiretroviral treatment adherence.
- The findings from this study have the potential to provide an understanding of key cultural and social determinants that must be addressed to increase treatment adherence and engagement in care among African Americans living with HIV.

Weaknesses

- None noted

INVESTIGATOR(S):

Strengths

- The research team has demonstrated success in recruiting African Americans into previous studies. This is noteworthy given the mistrust among this population as a result of how research was conducted among African Americans historically.

Weaknesses

- None noted

INNOVATION:

Strengths

- The use of a treatment advocacy intervention that is designed for African Americans living with HIV.
- The intervention integrates culturally relevant factors.

Weaknesses

- None noted

APPROACH:

Strengths

- The intervention sessions/activities are situated within a framework that provides structure, cultural relevance and logical sequence.
- A conceptual diagram and a table that describes the intervention is included which provides a linkage to stated hypotheses with statistical analysis.
- Previous studies conducted by the PI provide evidence that the various components are effective for treatment adherence.
- Booster sessions should enhance the overall effectiveness of the TA intervention.
- Randomization process detailed.
- This Important next step tests the TA intervention components in a comprehensive structured intervention.

Weakness

- There is a small possibility that control participants may be exposed to some of the TA components.
- A CBPR model/principles to be used to guide the research process is not included.
- A plan to evaluate the CBPR process is not included.

ENVIRONMENT:

Strengths

- The APLA setting where the study will be conducted is an appropriate location to test the TA intervention.

- Previous studies conducted by AIDS project Los Angeles (APLA) have demonstrated continuous commitment and support to community based research.

Weaknesses

- None noted

THE FOLLOWING REVIEW CRITERIA ARE NOT SCORED INDIVIDUALLY, BUT SHOULD BE CONSIDERED WHEN DETERMINING THE OVERALL IMPACT/PRIORITY SCORE.

Protections for Human Subjects:

Acceptable Risks and/or Adequate Protections

Data and Safety Monitoring Plan (Applicable for Clinical Trials Only):

Acceptable

Inclusion of Women, Minorities and Children:

G1A - Both Genders, Acceptable

M2A - Only Minority, Acceptable

C1A - Children Included, Acceptable

Vertebrate Animals:

Not Applicable (No Vertebrate Animals)

Resubmission:

- This resubmission of the application to support a randomized controlled trial (RCT) of an innovative culturally relevant treatment advocacy (TA) intervention for African Americans with HIV that targets cultural and social issues contributing to health disparities. The great majority of the items in the summary statement were clarified in the resubmission. Concerns about the lack of detail on recruitment and intervention was addressed and the need for a stronger conceptualization that ties intervention to outcome model to analysis was clarified and broadened where stated hypotheses and statistical tests are linked to a more refined conceptual diagram and intervention description.

BUDGET AND PERIOD OF SUPPORT:

Recommend as Requested

CRITIQUE 2:

Significance	1
Investigator(s)	1
Innovation	1
Approach	1
Environment	1

OVERALL IMPACT:

Strengths

- Overall, the PI and her team have responded well to the concerns of the previous review and have addressed sufficiently the significant issues that were raised. As a result, it is likely to have an important and significant impact on the targeted sample and by extension community of AA men who have adherence issues to HAART.
- The testing of the efficacy of an adherence intervention that specifically targets a community hardest hit by HIV and which has what appears to be a significant disparity in adherence (when compared to European Americans) is important and if the tailored intervention proves efficacious, the impact of the study on health for AA men and reducing disparity in adherence between AA and EA is significant

Weaknesses

- None

SIGNIFICANCE:

Strengths

- This resubmission is based on pilot work done by the PI that demonstrates that African American men are receptive to treatment advocacy (TA), and believe TA may reduce the disparity between AA men and European American men, vis a vis adherence to HAART
- The pilot data presented demonstrates that there is a health disparity around adherence behavior between AA and EA men.
- Since the AA community is so heavily over-represented in the HIV community, the evaluation of an intervention specifically tailored to improve adherence in AA men could potentially be significant if the intervention is demonstrated to be effective.

Weaknesses

- None

INVESTIGATOR(S):

Strengths

- Very strong research team that has a history of working together, particularly on the TA intervention
- Although the team is spread throughout the country, each brings unique and distinct skills to the overall project.

Weaknesses

- A shared workload plan would have been preferred– perhaps a timeline with who is doing what, when on the project to better understand the flow of the workload

INNOVATION:

Strengths

- The TA intervention itself, although not tested for its effectiveness in AA men, is very innovative. The domains, which the intervention focuses on, appear to be highly relevant and tailored to AA men and the researchers seem to have evolved them out of the community.
- Simply designing an intervention for adherence specifically for AA participants that takes cultural context into account is innovative, given the fact that few currently exist in the literature

Weaknesses

- None

APPROACH:

Strengths

- The use of CBPR is a good approach given the argument that in AA men specific cultural issues contribute to low adherence. This resubmission presents a more full description of the CBPR used for this proposed study.
- This resubmission addresses several of the criticisms from reviewers on its initial submission – specifically the PI provide a better description of the randomization process (sound), more detailed information on the culturally relevant aspects of TA are included (thoroughly discussed), hypotheses are made explicit (reasonable), the intervention is expanded with more sessions and a maintenance phase (appropriately)
- Table 1a is presents a solid description of the session components, goals and activities as well as connects proposed mediators.

Weaknesses

- None

ENVIRONMENT:

Strengths

- The environment at each institution where the investigators reside appears to be excellent for the successful completion of this proposed study.
- CBO's appear to be on board with this study, which bodes well for a successful partnership and completion of the study
- The familiarity with the proposed site of the study (AIDS Project LA) and the team's past experience with the site, make for an excellent environment in which to conduct the study.

Weaknesses

- None

THE FOLLOWING REVIEW CRITERIA ARE NOT SCORED INDIVIDUALLY, BUT SHOULD BE CONSIDERED WHEN DETERMINING THE OVERALL IMPACT/PRIORITY SCORE.

Protections for Human Subjects:

Acceptable Risks and/or Adequate Protections

- This proposal provides a detailed description of adequate protections for participants in this study. The risks appear to be low and potential problems adequately addressed

Data and Safety Monitoring Plan (Applicable for Clinical Trials Only):

Acceptable

- o The PI argues that an extensive monitoring plan is not necessary given the low risk involved in this study. This appears to be a reasonable argument and thus the plan appears to be acceptable.

Inclusion of Women, Minorities and Children:

G1A - Both Genders, Acceptable

M2A - Only Minority, Acceptable

C1A - Children and Adults, Acceptable

- This study targets specifically AA's thus minority only is acceptable. A small sample of women is included and participants in the 18 to 21 year old range are included, thus the proposal includes both children and adults.

Vertebrate Animals:

Not Applicable (No Vertebrate Animals)

Resubmission:

- The PI and her team have addressed sufficiently the previous reviewers' criticisms. The current proposal as it stands is very strong and likely to have a significant impact.

Budget and Period of Support:

Recommend as Requested

CRITIQUE 3:

Significance	1
Investigator(s)	1
Innovation	1
Approach	2
Environment	1

OVERALL IMPACT:

Strengths

- This is a resubmission of an R01 application. The PI was very responsive to previous critiques and a number of modifications have been made. In addition, additional details about critical components of study design have been added.
- The end product is a stronger application that would test a culturally-relevant treatment advocacy (TA) intervention for African Americans with HIV.
- The innovative TA targets social and cultural issues that contribute to poor HIV treatment behaviors.
- Significance is high, as this would be the first systematic evaluation of TA, which enjoys widespread use by community agencies all over the country since the HIV epidemic began.

Weaknesses

- Weaknesses were minor and focused on study assessments and specific measures used to evaluate results from the clinical trial.

- In particular, the absence of standardized measures of substance use/abuse and associated problems and the lack of synchronicity between text and citations for this domain was of some concern

SIGNIFICANCE:

Strengths

- Few culturally relevant interventions to promote adherence with ART treatment have been tested using clinical trials methods
- Present study will experimentally evaluate an innovative, culturally-relevant treatment advocacy (TA) intervention created to specifically focus on the cultural and social issues contributing to health disparities for African Americans (AAs) with HIV.
- If effective, the enhanced TA intervention could contribute to improvement in health outcomes for AAs with HIV and ultimately reduce disparities

Weaknesses

- None noted

INVESTIGATOR(S):

Strengths

- The PI and Co-Investigators are well-qualified to conduct the proposed research
- They possess the practical/experiential as well as scientific skills and background needed for this research

Weaknesses

- None noted

INNOVATION:

Strengths

- While TA interventions have been maintained and sustained across the USA (and are believed to play a vital role in assisting HIV patients with care access and retention), no RCTs have been published evaluating TAs, and to-date little attention has focused on unique issues of AA patients.
- The proposed clinical trial is unique in several ways. In particular, the TA intervention to be evaluated was developed to specifically focus on cultural issues linked to non-adherence in AA participants (e.g., HIV misconceptions, discrimination, medical mistrust)

Weaknesses

- None noted

APPROACH:

Strengths

- In addition to positive aspects of the research design highlighted in the previous review of the application, revised proposal has been strengthened in several ways
- Background section now highlights fact that the TA intervention in the proposal was developed specifically for AAs and takes into consideration their unique cultural context of this target group

- Conceptual model of mechanisms by which the culturally-relevant TA intervention might improve health outcomes (and thereby reduce disparities) is now featured in the proposal.
- Details about training of relevant staff has been added to the application

Weaknesses

- Since substance abuse/dependence is associated with lower rates of adherence with ART and substance use was highlighted as a culturally-relevant issue for AAs, it was surprising to see so few ACASI (Audio computer-assisted self-interview) measures at baseline and follow-up focused on alcohol/drug use, problems and treatment.
- For drug use, the only measure described in any detail was an index based on frequency of recent drug use (past 30 days). Unfortunately, the two articles cited in support of this index were published in 1990 and appear to be specific to alcohol. Thus, no standardized measure of drug use, problems (including legal issues), and intervention services (treatment) was found.

ENVIRONMENT:

Strengths

- Outstanding

Weaknesses

- None noted

THE FOLLOWING REVIEW CRITERIA ARE NOT SCORED INDIVIDUALLY, BUT SHOULD BE CONSIDERED WHEN DETERMINING THE OVERALL IMPACT/PRIORITY SCORE.

Protections for Human Subjects:

Acceptable Risks and/or Adequate Protections

Data and Safety Monitoring Plan (Applicable for Clinical Trials Only):

Acceptable

Inclusion of Women, Minorities and Children:

G1A - Both Genders, Acceptable

M2A - Only Minority, Acceptable

C1A - Children and Adults, Acceptable

Vertebrate Animals:

Not Applicable (No Vertebrate Animals)

BUDGET AND PERIOD OF SUPPORT:

Recommend as Requested

THE FOLLOWING RESUME SECTIONS WERE PREPARED BY THE SCIENTIFIC REVIEW OFFICER TO SUMMARIZE THE OUTCOME OF DISCUSSIONS OF THE REVIEW COMMITTEE ON THE FOLLOWING ISSUES:

PROTECTION OF HUMAN SUBJECTS (Resume): Applicable, Acceptable

INCLUSION OF WOMEN PLAN (Resume): Acceptable G1A

INCLUSION OF MINORITIES PLAN (Resume): Acceptable M2A

INCLUSION OF CHILDREN PLAN (Resume): Acceptable C1A

COMMITTEE BUDGET RECOMMENDATIONS: The budget was recommended as requested.

SCIENTIFIC REVIEW OFFICER'S NOTES: Consistent with the NIH policy framework, the NIMHD R01 resubmission (amended – A1) applications from new and established investigators must abide by receipt dates specified on subsequent competing RFAs. Alternatively, R01 resubmission (amended – A1) applications to the Center for Scientific Review (CSR) study sections from new investigators may be submitted on a special receipt date for review in the very next review cycle. For more details, see notice in the NIH Guide for Grants and Contracts: http://grants1.nih.gov/grants/guide/notice-files/NOT-OD-07-083.html.

You can contact the NIH Program Officer whose name appears on the upper left hand corner of page one of this Summary Statement for information about probable funding or whether your application merits resubmission. The program officer can also help you decide whether the changes and improvements necessary to address the weaknesses noted in the reviewers' critiques could be accomplished in the relatively short time available. You are also strongly advised to seek input from mentors, your Department chair, etc.

You may choose to take more time to resubmit your application. For those directed to CSR, you should prepare the resubmission for the normal dates specified in the following table: http://grants1.nih.gov/grants/funding/submissionschedule.htm. NIMHD would only accept resubmission applications on receipt dates listed on competing RFAs.

NIH has modified its policy regarding the receipt of resubmissions (amended applications). See Guide Notice NOT-OD-10-080 at http://grants.nih.gov/grants/guide/notice-files/NOT-OD-10-080.html.
The impact/priority score is calculated after discussion of an application by averaging the overall scores (1-9) given by all voting reviewers on the committee and multiplying by 10. The criterion scores are submitted prior to the meeting by the individual reviewers assigned to an application, and are not discussed specifically at the review meeting or calculated into the overall impact score. For details on the review process, see http://grants.nih.gov/grants/peer_review_process.htm#scoring.

MEETING ROSTER

National Center on Minority Health and Health Disparities Special Emphasis Panel
NATIONAL CENTER ON MINORITY HEALTH AND HEALTH DISPARITIES
R01
ZMD1 MLS (01) 2
December 15, 2010 - December 16, 2010

CHAIRPERSON

WUBAH, DANIEL A., PHD
VICE PRESIDENT AND DEAN FOR UNDERGRAD EDUCATION
VIRGINIA TECH
BLACKSBURG, VA 24061

MEMBERS

ABDEL-RAHMAN, ABDEL A, PHD
DISTINGUISHED PROFESSOR OF PHARMACOLOGY
DEPARTMENT OF PHARMACOLOGY
EAST CAROLINA UNIVERSITY SCHOOL OF MEDICINE
GREENVILLE, NC 27834

AREAN, PATRICIA A., PHD
PROFESSOR
DEPARTMENT OF PSYCHIATRY
UNIVERSITY OF CALIFORNIA, SAN FRANCISCO
SAN FRANCISCO, CA 94143

CANINO, GLORISA J., PHD
PROFESSOR AND DIRECTOR
DEPARTMENT OF PEDIATRICS
BEHAVIORAL SCIENCES RESEARCH INSTITUTE
UNIVERSITY OF PUERTO RICO
SAN JUAN, PR 009365067

CARRASQUILLO, OLVEEN , MD, PHD
ASSOCIATE PROFESSOR
CHIEF DIVISION OF GENERAL MEDICINE
UNIVERSITY OF MIAMI
MILLER SCHOOL OF MEDICINE
MIAMI, FL 33136

CLARK, MELISSA A., PHD
ASSOCIATE PROFESSOR OF COMMUNITY HEALTH, OBSTETRICS AND GYNECOLOGY
BROWN UNIVERSITY
PROVIDENCE, RI 02903

DENNIS, BETTY P., RN, BSN, DRPH
PROFESSOR AND DEAN
DIVISION OF NURSING
DILLARD UNIVERSITY
NEW ORLEANS, LA 70122

DOORENBOS, ARDITH Z., BSN, PHD
ASSOCIATE PROFESSOR
BIOBEHAVIORAL NURSING & HEALTH SYSTEMS
SCHOOL OF NURSING, GLOBAL HEALTH, SCHOOL OF MED
UNIVERSITY OF WASHINGTON
SEATTLE, WA 98195

ESTRADA, ANTONIO L., PHD
PROFESSOR
DIRECTOR, MEXICAN AMERICAN STUDIES AND RESEARCH CENTER
COLLEGE OF SOCIAL AND BEHAVIORAL SCIENCES
UNIVERSITY OF ARIZONA
TUCSON, AZ 857210023

EVERSON-ROSE, SUSAN A., MPH, PHD
ASSOCIATE PROFESSOR
DEPARTMENT OF MEDICINE
RESEARCH CORE, CENTER FOR HEALTH EQUITY
UNIVERSITY OF MINNESOTA
MINNEAPOLIS, MN 55414

FINCH, BRIAN K., PHD
PROFESSOR
DEPARTMENT OF SOCIOLOGY AND PUBLIC HEALTH
SAN DIEGO STATE UNIVERSITY
SAN DIEGO, CA 92182

GOLUB, ANDREW L., PHD
PRINCIPAL INVESTIGATOR, NDRI
INSTITUTE FOR SPECIAL POPULATIONS RESEARCH NDRI
BURLINGTON , VT 05401

GOZAL, LEILA KHEIRANDISH, MD
ASSOCIATE PROFESSOR OF PEDIATRICS
DIRECTOR OF CLINICAL SLEEP RESEARCH
DEPARTMENT OF PEDIATRICS
THE UNIVERSITY OF CHICAGO
CHICAGO, IL 60637

GREINER, K ALLEN, MPH, MD
ASSOCIATE PROFESSOR OF MEDICINE
UNIVERSITY OF KANSAS
DIR.OF RESEARCH AND RURAL PRIMARY CARE RESEARCH PROGRAM
KANSAS CITY, KS 66160

HAHN, ELIZABETH A., MA
ASSOCIATE PROFESSOR
NORTHWESTERN UNIV SCH OF MEDICINE
FEINBERG SCHOOL OF MEDICINE
CHICAGO, IL 60611

HOLM, KARYN , FAAN, RN, PHD
PROFESSOR
DEPARTMENT OF NURSING
VINCENT DE PAUL PROFESSOR
DEPAUL UNIVERSITY
CHICAGO, IL 60614

JONES, WARREN A., MD
DISTINGUISHED PROFESSOR OF HEALTH POLICY
PROFESSOR OF FAMILY MEDICINE
UNIVERSITY OF MISSISSIPPI MEDICAL CENTER
JACKSON, MS 39216

KERSHAW, JOSEPHINE M., PHD
ASSOCIATE PROFESSOR
DIVISION OF HEALTH CARE MANAGEMENT
UNIVERSITY OF FINDLAY
FINDLAY, OH 45840

KIM, MIYONG T., FAAN, PHD
PROFESSOR AND CHAIR
DEPARTMENT OF HEALTH SYSTEMS AND OUTCOMES
SCHOOL OF NURSING
JOHNS HOPKINS UNIVERSITY
BALTIMORE, MD 21205

KIMM, SUE Y.S., MPH, MD
ADJUNCT PROFESSOR OF EPIDEMIOLOGY
DEPARTMENT OF INTERNAL MEDICINE
UNIVERSITY OF NEW MEXICO
ALBUQUERQUE, NM 87131

KULIS, STEPHEN S. PH.D.
PROFESSOR
SOUTHWEST INTERDISCIPLINARY RESEARCH CENTER
ARIZONA STATE UNIVERSITY
PHOENIX , AZ 85004-069

LOWE, JOHN R. PHD, BSN
ASSOCIATE PROFESSOR
COLLEGE OF NURSING
FLORIDA ATLANTIC UNIVERSITY, DAVIE CAMPUS
DAVIE, FL 33314

MANNE, UPENDER , PHD
ASSOCIATE PROFESSOR
DEPARTMENT OF PATHOLOGY
UNIVERSITY OF ALABAMA
BIRMINGHAM, AL 35294

MCCORMACK BROWN, KELLI CHES, PHD
PROFESSOR AND ASSOCIATE DEAN
COLLEGE OF HEALTH & HUMAN PERFORMANCE
UNIVERSITY OF FLORIDA
GAINESVILLE, FL 32611

MCELRATH, THOMAS FREDERICK, MD, PHD
ASSISTANT PROFESSOR
BRIGHAM AND WOMEN'S HOSPITAL,
DIVISION OF MATERNAL-FETAL MEDICINE
OBSTETRICS/GYNECOLOGY: MATERNAL-FETAL
MEDICINE
BOSTON , MA 02115

MURPHY, MAUREEN E., RN, PHD
ASSOCIATE PROFESSOR
PROGRAM IN MOLECULAR AND TRANSLATIONAL
MEDICINE
FOX CHASE CANCER CENTER
PHILADELPHIA, PA 19111

MURTAUGH, MAUREEN A., PHD
ASSOCIATE PROFESSOR
DEPARTMENT OF INTERNAL MEDICINE
SCHOOL OF MEDICINE
THE UNIVERSITY OF UTAH
SALT LAKE CITY, UT 84108

NIES, MARY A., FAAN, RN, BSN, PHD
CAROL GROTNES BELK ENDOWED CHAIR IN NURSING &
PROFESSOR
ADJUNCT PROFESSOR PUBLIC HEALTH SCIENCES
COLLEGE OF HEALTH AND HUMAN SERVICES
UNIVERSITY OF NORTH CAROLINA AT CHARLOTTE
CHARLOTTE, NC 28223

OPARA, EMMANUEL C., PHD
PROFESSOR OF REGENERATIVE MEDICINE
INSTITUTE FOR REGENERATIVE MEDICINE AND CNTR.
FOR
DIABETES RESEARCH
WAKE FOREST UNIVERSITY SCHOOL OF MEDICINE
10 WEST 32ND ST, E1-116
WINSTON-SALEM, NC 27157

PANDEY, JANARDAN P, PHD
PROFESSOR OF MICROBIOLOGY IMMUNOLOGY
MEDICAL UNIV OF SOUTH CAROLINA
CHARLESTON, SC 29425

REIJO PERA, RENEE A, PHD
ASSOCIATE PROFESSOR
CENTER FOR HUMAN EMBRYONIC
STEM CELL RESEARCH
DEPARTMENT OF OBSTETRICS AND GYNECOLOGY
STANFORD UNIVERSITY SCHOOL OF MEDICINE
STANFORD UNIVERSITY SCHOOL OF MEDICINE
PALO ALTO, CA 94304

ROYAK-SCHALER, RENEE PHD, MED
ASSOCIATE PROFESSOR
DIRECTOR, MPH PROGRAM
DEPARTMENT OF EPIDEMIOLOGY AND
PREVENTIVE MEDICINE
UNIVERSITY OF MARYLAND SCHOOL OF MEDICINE
BALTIMORE, MD 21201

SAMBAMOORTHI, USHA , PHD
PROFESSOR
SCHOOL OF PHARMACY
WEST VIRGINIA UNIVERSITY
MORGANTOWN, WV 26501

SANDERS-PHILLIPS, KATHY , PHD
PROFESSOR OF PEDIATRICS AND CHILD HEALTH
DEPARTMENT OF PEDIATRICS
HOWARD UNIVERSITY
WASHINGTON, DC 20001

SCHOLL, THERESA O. PHD, MPH
PROFESSOR, OBSTETRICS AND GYNECOLOGY
DEPARTMENT OF OBSTETRICS AND GYNECOLOGY
UNIVERSITY OF MEDICINE AND DENTISTRY OF NEW
JERSEY
STRATFORD, NJ 08084

SEGAL, MARY E, PHD
DIRECTOR OF RESEARCH
INSTITUTE ON DISABILITIES
TEMPLE UNIVERSITY
PHILADELPHIA, PA 19104

SHEPPARD, VANESSA B., PHD
ASSISTANT PROFESSOR
LOMBARDI CANCER CENTER
GEORGETOWN UNIVERSITY MEDICAL CENTER
WASHINGTON, DC 20007

STECK, SUSAN E, PHD
DEPT OF EPIDEMIOLOGY AND BIOSTATISTICS
CANCER PREVENTION AND CONTROL PROGRAM
UNIV OF SOUTH CAROLINA, ARNOLD SCH OF PUBLIC HLTH
COLUMBIA, SC 29201

STRAYHORN, GREGORY , MD, PHD
PROFESSOR
DEPARTMENT OF FAMILY MEDICINE
MOREHOUSE SCHOOL OF MEDICINE
ATLANTA, GA 30308

SVIKIS, DACE S., PHD
PROFESSOR
DEPARTMENTS OF PSYCHOLOGY, PSYCHIATRY AND OB/GYN
COLLEGE OF HUMANITIES AND SCIENCES AND
SCHOOL OF MEDICINE, MEDICAL COLLEGE OF VIRGINIA
VIRGINIA COMMONWEALTH UNIVERSITY
RICHMOND, VA 232980343

VOSVICK, MARK A., PHD
ASSOCIATE PROFESSOR
CLINICAL HEALTH PSYCHOLOGY AND BEHAVORIAL MEDICINE
PSYCHOLOGY DEPARTMENT
UNIVERSITY OF NORTH TEXAS
DENTON, TX 76203

YOUNG, BESSIE A, MD
ASSOCIATE PROFESSOR
DEPARTMENT OF EPIDEMIOLOGY
UNIVERSITY OF WASHINGTON
SEATTLE, WA 98108

SCIENTIFIC REVIEW ADMINISTRATOR

LAUDE-SHARP, MARYLINE , PHD
SCIENTIFIC REVIEW OFFICER
NATIONAL INSTITUTES ON MINORITY HEATLH
AND HEALTH DISPARITIES
NATIONAL INSTITUTES OF HEALTH
BETHESDA, MD 20892

GRANTS TECHNICAL ASSISTANT

LIPKIND, JOANNE , MS
EXTRAMURAL SUPPORT ASSISTANT
NATIONAL INSTITUTE ON MINORITY HEALTH
AND HEALTH DISPARITIES
NATIONAL INSTITUTES OF HEALTH
BETHESDA, MD 20892

Consultants are required to absent themselves from the room during the review of any application if their presence would constitute or appear to constitute a conflict of interest.
